# Supplementary figures and images for: Epidemiological survey of serum titers from adults against various Gram-negative bacterial V-antigens
Source: PLoS One. 2020 Mar 10;15(3):e0220924. doi: 10.1371/journal.pone.0220924 (PMC7064248; doi:10.1371/journal.pone.0220924)

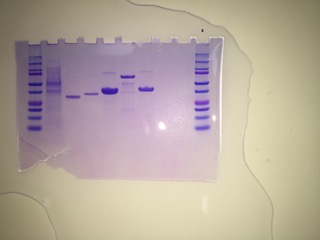

Supplement: S1 Fig — (JPEG) [file pone.0220924.s001.jpeg]
