# Supplementary material for: Epidemiological survey of serum titers from adults against various Gram-negative bacterial V-antigens
Source: PLoS One. 2020 Mar 10;15(3):e0220924. doi: 10.1371/journal.pone.0220924 (PMC7064248; doi:10.1371/journal.pone.0220924)
Supplement: Attachment — Submitted filename: 11-3-19 plos one review.docx [file pone.0220924.s002.docx]

Comments

In this manuscript, Kinoshita et al tried to establish serum anti-V antibody titer as method for the epidemiological investigations on various pathogenic Gram-negative bacteria. Authors developed an enzyme-linked immuno-sorbent assay to measure titers against each V antigen in the sera collected from 186 adult volunteers. However, the manuscript is identified with a rather confusing experimental approaches for epidemiological investigation of Gram-negative bacterial pathogens. The application of purified V antigens (PcrV from *Pseudomonas aeruginosa*, LcrV from *Yersinia*, LssV from *Photorhabdus luminescens*, AcrV from *Aeromonas salmonicida* and VcrV from *Vibrio parahaemolyticus*) and their cross reactivity in both human and mouse serum samples demonstrating the inability of this system to determine accurately the bacterium elicited antigenicity in human samples. The manuscript is also suffering with the complete lack of standard or control sample in entire experiments and that raised serious question against reproducibility of work.
