# Supplementary material for: Epidemiological survey of serum titers from adults against various Gram-negative bacterial V-antigens
Source: PLoS One. 2020 Mar 10;15(3):e0220924. doi: 10.1371/journal.pone.0220924 (PMC7064248; doi:10.1371/journal.pone.0220924)
Supplement: Attachment — Submitted filename: ResponseToComments.docx [file pone.0220924.s003.docx]

Journal Requirements:

1. When submitting your revision, we need you to address these additional requirements.
Please ensure that your manuscript meets PLOS ONE's style requirements, including those for file naming. The PLOS ONE style templates can be found at
<http://www.journals.plos.org/plosone/s/file?id=wjVg/PLOSOne_formatting_sample_main_body.pdf> and <http://www.journals.plos.org/plosone/s/file?id=ba62/PLOSOne_formatting_sample_title_authors_affiliations.pdf>

[Response]

I followed the above guidance.

2. PLOS ONE now requires that authors provide the original uncropped and unadjusted images underlying all blot or gel results reported in a submission’s figures or Supporting Information files. This policy and the journal’s other requirements for blot/gel reporting and figure preparation are described in detail at [https://journals.plos.org/plosone/s/figures#loc-blot-and-gel-reporting-requirements](https://journals.plos.org/plosone/s/figures#loc-blot-and-gel-reporting-requirements) and [https://journals.plos.org/plosone/s/figures#loc-preparing-figures-from-image-files](https://journals.plos.org/plosone/s/figures#loc-preparing-figures-from-image-files). When you submit your revised manuscript, please ensure that your figures adhere fully to these guidelines and provide the original underlying images for all blot or gel data reported in your submission. See the following link for instructions on providing the original image data: [https://journals.plos.org/plosone/s/figures#loc-original-images-for-blots-and-gels](https://journals.plos.org/plosone/s/figures#loc-original-images-for-blots-and-gels).

In your cover letter, please note whether your blot/gel image data are in Supporting Information or posted at a public data repository, provide the repository URL if relevant, and provide specific details as to which raw blot/gel images, if any, are not available. Email us at [plosone@plos.org](mailto:plosone@plos.org) if you have any questions.

[Response]

We provide an original uncropped and unadjusted SDS-PAGE image of Fig.1, and wrote it in our cover letter for this resubmission.

3. Please provide additional details regarding participant consent. In the ethics statement in the Methods and online submission information, please ensure that you have specified (1) whether consent was suitably informed and (2) what type you obtained (for instance, written or verbal). If your study included minors under age 18, state whether you obtained consent from parents or guardians. If the need for consent was waived by the ethics committee, please include this information.

[Response]

We added the following:

Page 8, line 104 (page 8, line 139 of tracked_change_version): “As a non-interventional and non-invasive retrospective observational study, the need for consent was waived by the ethics committee.”

4. To comply with PLOS ONE submissions requirements, please provide methods of sacrifice in the Methods section of your manuscript.

[Response]

We added the following:

Page 11, line 152 (page 11, line 214 of tracked_change_version):” …., the immunized mice were euthanized with a high dose intraperitoneal injection of sodium pentobarbital,”

5. We noticed you have some minor occurrence(s) of overlapping text with the following previous publication(s), which needs to be addressed:
<https://doi.org/10.1111/1348-0421.12353>
<https://doi.org/10.1111/1348-0421.12147>
<https://iai.asm.org/content/iai/65/2/446.full.pdf>

In your revision ensure you cite all your sources (including your own works), and quote or rephrase any duplicated text outside the Methods section. Further consideration is dependent on these concerns being addressed.

[Response]

We provided the correct reference numbers in all the sentences which we cited.

6. Thank you for stating the following in the Competing Interests section:

I have read the journal's policy and the authors of this manuscript have the following competing interests: T. Sawa has patents associated with PcrV immunization (World Patent No. WO0033872; European Patent No. EP1049488; U.S. Patent No. 6309651; U.S. Patent No. 6827935, and Japan Patent No. 2017-020501). Until 2011, T. Sawa received a patent fee from the Regents of the University of California related to the development of a therapeutic monoclonal antibody at KaloBios Pharmaceutical. There are no current financial relationships existing with any organization associated with this study.

Please confirm that this does not alter your adherence to all PLOS ONE policies on sharing data and materials, by including the following statement: "This does not alter our adherence to  PLOS ONE policies on sharing data and materials.” (as detailed online in our guide for authors <http://journals.plos.org/plosone/s/competing-interests>).  If there are restrictions on sharing of data and/or materials, please state these. Please note that we cannot proceed with consideration of your article until this information has been declared.

Please include your updated Competing Interests statement in your cover letter; we will change the online submission form on your behalf.

Please know it is PLOS ONE policy for corresponding authors to declare, on behalf of all authors, all potential competing interests for the purposes of transparency. PLOS defines a competing interest as anything that interferes with, or could reasonably be perceived as interfering with, the full and objective presentation, peer review, editorial decision-making, or publication of research or non-research articles submitted to one of the journals. Competing interests can be financial or non-financial, professional, or personal. Competing interests can arise in relationship to an organization or another person. Please follow this link to our website for more details on competing interests: <http://journals.plos.org/plosone/s/competing-interests>

[Response]

We added the sentence as follows, in the section Conflict of Interest:

This does not alter our adherence to PLOS ONE policies on sharing data and materials.

Responses to Reviewer comments:

Reviewer #1: In this manuscript, Kinoshita et al tried to establish serum anti-V antibody titer as method for the epidemiological investigations on various pathogenic Gram-negative bacteria. Authors developed an enzyme-linked immuno-sorbent assay to measure titers against each V antigen in the sera collected from 186 adult volunteers. However, the manuscript is identified with a rather confusing experimental approaches for epidemiological investigation of Gram-negative bacterial pathogens. The application of purified V antigens (PcrV from Pseudomonas aeruginosa, LcrV from Yersinia, LssV from Photorhabdus luminescens, AcrV from Aeromonas salmonicida and VcrV from Vibrio parahaemolyticus) and their cross reactivity in both human and mouse serum samples demonstrating the inability of this system to determine accurately the bacterium elicited antigenicity in human samples. The manuscript is also suffering with the complete lack of standard or control samples in entire experiments and that raised serious question against reproducibility of work.
Authors must include the closely related species and or strains of all five bacteria used in this study to validate it further.

[Response]

We understand some of the concerns raised by the reviewer. There is no standard human anti-V-antigen IgG except for human monoclonal anti-PcrV IgG. Therefore, after optimization of the ELISA system for anti-PcrV titers using the anti-PcrV monoclonal standard [ref 32], the optical density measured under a consistent condition with the same secondary antibody was used to evaluate the titers. We do not agree with the reviewer’s suggestion to use closely related species or strains of all five bacteria. We used five recombinant V-antigens from five different species. Among the same species, variation in the primary sequences of V-antigens is quite low. Thus, there is no reason to include closely related species or strains. However, in an additional experiment, we included *P. aeruginosa* OprF as an irrelevant non-V-antigen protein that has no similarity with the tested V-antigens in terms of primary sequences. As a result, in the inhibition ELISA using OprF, preincubation with OprF did not affect the optical density measured for anti-V-antigen titers, whereas preincubation with LssV significantly decreased anti-V-antigen titers for ArcV and LssV as evidence of cross-antigenicity (new Fig. 5 Inhibition ELISA).

As the reviewer pointed out, there is no standard sample for titer measurement of anti-LcrV, anti-LssV, anti-VcrV, and anti-AcrV. Except for human monoclonal anti-PcrV IgG mAb 6F5 as a standard to measure the anti-PcrV titer [32], there is no human standard anti-V-antigen IgG. Therefore, after optimization of the ELISA system for anti-PcrV titers using the mAb 6F5 standard [32], the optical density measured under a consistent condition with the same secondary antibody was used to evaluate the titers. Accordingly, we have added the following text.

Page 14, line 202 (page 13, line 282 of tracked_change_version): Next, the inhibition ELISA was performed to show cross-antigenicity among V-antigens. As an irrelevant non-crossreactive protein, we prepared *P. aeruginosa* recombinant OprF using the same *E.coli*-derived recombinant protein construction system. The sequence alignment scores obtained from ClustalW between OprF and V-antigens were 9.8–11.4, whereas those among V-antigens were from 21.3 (between VcrV and AcrV) to 48.3 (between LcrV and LssV). Three human sera diluted 1000× were preincubated with either recombinant LssV or recombinant OprF overnight. Then, preprocessed serum titers against each V-antigen were measured in comparison with the titer levels of the original sera (**Fig. 5**). As a result, preincubation with OrpF did not affect the specific titer levels. However, preincubation with LssV decreased the titer levels compared with the titer levels of the original sera (**p*<0.05 for AcrV, VcrV, and LssV).

Page 9, line 130 (page 9, line 178 of tracked_change_version): Except for human monoclonal anti-PcrV IgG mAb 6F5 as a standard to measure the anti-PcrV titer [32], there is no human anti-V-antigen IgG. Therefore, after optimization of the ELISA system for anti-PcrV titers using the mAb 6F5 standard [32], the OD measured under a consistent condition with the same secondary antibody was used to evaluate the titers.

Reviewer #2: Major comments:
1) Figure 4A doesn't match to the written part that describe it and actually it is identical to figure 7A. Please correct the figure.

[Response]

We have corrected the error pointed out by the reviewer. In the revised manuscript, Figure 4A is the correct figure.

2) There is no statistical method section in the Materials and Methods part so it is impossible to determine statistical significance, power analysis etc. Please include such a section with the relevant statistical analyses.

[Response]

As suggested, we have added a new section “Statistical analysis” in the Materials and methods.

3) Regarding the heat maps, please provide a valid statistical/numerical information that defines the various colors. I'm sure that this data is provided by the relevant analysis tools. Please include a short description also in the figure legend.

[Response]

We have added numerical information about the color heat maps in figures. We have added the methodology to construct the heat maps in the newly added section “Phylogenetic and cluster analyses“ and the explanation of the color-index in each figure legend.

Minor comments:
1)The sentence in line 273 is not clear.

[Response]

As pointed out by the reviewer, the description of this sentence was unclear. We have rewritten the sentence as follows.

Page 20, line 316 (page 20, line 525 of tracked_change_version): Regarding the blocking antibody recognizing the three-dimensional conformational epitopes and not the primary amino acid sequences, not only the serum titer levels, but also the specific components that bind to the specific blocking regions in V-antigen molecules may be important to prevent the pathogenesis associated with TTSS virulence.

2)Line 30; a dot is missing after the 0.7

[Response]

The error has been corrected.

3)Line 117; please correct the 0.0M to a valid value.
It has been changed to “0.05 M”.

4)Line 119; please correct the 200mL to 200μL
[Response]

It has been changed to “200 μL”.

5)Line 121; please correct the 100mL/well to 100μL/well
[Response]

It has been changed to “100 μL”.

6)line 125; please correct the 100mL/well to 100μL/well
[Response]

It has been changed to “100 μL”.

7)line 224; please correct "..carboxyl domains.." to carboxyl-terminal domains
[Response]

The error has been corrected.

Reviewer #3: This paper by Kinoshita et. al. looks into the antibody response to various bacterial V-antigens in a cohort of healthy adults. They find that many people have various antibody responses to the V-antigens and that many of these antibodies may cross-react. The data is sound and the authors are careful not to overstate their findings. I do have a few comments however;

1. It is not made clear what percent of people have reactive antibodies to each protein. This could be a percent of patients over a specified cutoff. You already have both ‘red’ and ‘orange’ cutoffs so these could be used.

[Response]

On the basis of our previous results published in ref 18, we have added our speculation about the sufficient levels at which we can anticipate the protective effects because it is difficult to determine the cut-off levels.

Page 24, line 398 (page 24, line 640 of tracked_change_version): In this study, among the 198 volunteer-derived sera [32], the top 10 high titer sera against PcrV (greater than or equivalent to 0.5 at OD 450 nm) were used. Although we have human monoclonal IgG specific against PcrV as a standard for anti-PcrV measurement, no such human standard IgG against the other four V-antigens are available to date. It is difficult at this time to clearly evaluate where the level of protection is sufficient, but as many as 5% of the volunteers showed protective levels in our previous study [18], and the cross-antigenicity among the five V-antigens probably has a certain level of clinical significance in human immunity.

2. Both the correlations in Figure 3 and the mice vaccination studies strongly suggest the presence of some cross-reactive antibodies in the human sera. This would be fairly easy to prove in the human sera by absorption experiments. Take a human sera with responses to multiple proteins- adsorb against the highest reacting protein and determine if this also removes response to the other V-antigens.

[Response]

As the reviewer suggested, we have performed the additional experiment. We conducted an inhibition ELISA (sometimes called a competitive ELISA) to distinguish the levels of specific binding from the levels due to non-specific protein bindings. For high titer sera, we preincubated with either recombinant OprF (a *Pseudomonas aeuriginosa* surface antigen protein that is irrelevant to the V-antigen) or recombinant LssV and then measured the specific anti-V-antigen titers. The results are shown in new Fig. 5.

Page 14, line 202 (page 13, line282 of tracked_change_version): Next, the inhibition ELISA was performed to show cross-antigenicity among V-antigens. As an irrelevant non-crossreactive protein, we prepared *P. aeruginosa* recombinant OprF using the same *E.coli*-derived recombinant protein construction system. The sequence alignment scores obtained from ClustalW between OprF and V-antigens were 9.8–11.4, whereas those among V-antigens were from 21.3 (between VcrV and AcrV) to 48.3 (between LcrV and LssV). Three human sera diluted 1000× were preincubated with either recombinant LssV or recombinant OprF overnight. Then, preprocessed serum titers against each V-antigen were measured in comparison with the titer levels of the original sera (**Fig. 5**). As a result, preincubation with OrpF did not affect the specific titer levels. However, preincubation with LssV decreased the titer levels compared with the titer levels of the original sera (**p*<0.05 for AcrV, VcrV, and LssV).

3. Figure 5 would be easier to interpret if it was a graph or heat map of the response levels instead of a photo of the plate. I can’t tell by eye if one well is more blue than another.

[Response]

In accordance with the reviewer comment, we have omitted the previous Fig. 5.

4. Lines 244-248 the authors state that the amino-acid sequences phylogeny in Fig 7 do not match the serum trees in Fig 4A. To my eye they do match pretty well especially 7A and 7B. In both LssV is closest to LcrV and VcrV is furthest away. Can the authors clarify their statement?

[Response]

We apologize for our error. In Fig 4A, we mistakenly used Fig. 7A. We have corrected the error.

Comments

In this manuscript, Kinoshita et al tried to establish serum anti-V antibody titer as method for the epidemiological investigations on various pathogenic Gram-negative bacteria. Authors developed an enzyme-linked immuno-sorbent assay to measure titers against each V antigen in the sera collected from 186 adult volunteers. However, the manuscript is identified with a rather confusing experimental approaches for epidemiological investigation of Gram-negative bacterial pathogens. The application of purified V antigens (PcrV from *Pseudomonas aeruginosa*, LcrV from *Yersinia*, LssV from *Photorhabdus luminescens*, AcrV from *Aeromonas salmonicida* and VcrV from *Vibrio parahaemolyticus*) and their cross reactivity in both human and mouse serum samples demonstrating the inability of this system to determine accurately the bacterium elicited antigenicity in human samples. The manuscript is also suffering with the complete lack of standard or control sample in entire experiments and that raised serious question against reproducibility of work.

[Response]

As the reviewer pointed out, there is no standard sample for titer measurement of anti-LcrV, anti-LssV, anti-VcrV, and anti-AcrV. Except for human monoclonal anti-PcrV IgG mAb 6F5 as a standard to measure the anti-PcrV titer [32], there is no human standard anti-V-antigen IgG. Therefore, after optimization of the ELISA system for anti-PcrV titers using the mAb 6F5 standard [32], the optical density measured under a consistent condition with the same secondary antibody was used to evaluate the titers. We added the description about it in Page 9, line 130, as follows:

“Except for human monoclonal anti-PcrV IgG mAb 6F5 as a standard to measure the anti-PcrV titer [32], there is no human anti-V-antigen IgG. Therefore, after optimization of the ELISA system for anti-PcrV titers using the mAb 6F5 standard [32], the OD measured under a consistent condition with the same secondary antibody was used to evaluate the titers.”

Accordingly, we have added the following text. In addition, to confirm the cross-antigenicity, we added an additional experiment (inhibition ELISA with irrelevant protein OprF), and demonstrated the result in the new Fig 5.
