# Supplementary material for: Epidemiological survey of serum titers from adults against various Gram-negative bacterial V-antigens
Source: PLoS One. 2020 Mar 10;15(3):e0220924. doi: 10.1371/journal.pone.0220924 (PMC7064248; doi:10.1371/journal.pone.0220924)
Supplement: Attachment — Submitted filename: Response to Reviewers_2nd.docx [file pone.0220924.s004.docx]

[Response to Reviewers’ comments]

Review Comments to the Author
Please use the space provided to explain your answers to the questions above. You may also include additional comments for the author, including concerns about dual publication, research ethics, or publication ethics. (Please upload your review as an attachment if it exceeds 20,000 characters)

Reviewer #1: Authors have modified the manuscript as per the comments. However, revised manuscript is still having few grammatical mistakes, which need to be addressed before final submission.
[Response to the comment]

We fixed the following grammatical errors.

Page 4, line 50: the virulence associated -> the virulence-associated

Page 6, line 74: correlated and -> correlated, and

Page 6, line 87: multicloning site -> multiple cloning site

Page 8, line 108: March 2013 participated -> March 2013, participated

Page 9, line 128: a typo error: inhibiton -> inhibition

Page 11, line 153: by ELISAs as described -> by ELISAs, as described

Page 11, line 161: method and rooted trees -> method, and rooted trees

Page 12, line 176: regression analysis -> a regression analysis

Page 12, line 180: as considered statistically significant. -> considered statistically significant.

Page 13, line 204: coefficient 0.84 -> coefficient of 0.84

Page 14, line 226: unique profile -> a unique profile

Page 15, line 235: method -> method,

Page 15, line 240: method -> method,

Page 17, line 278: additional sequence -> an additional sequence

Page 22, line 357: a challenge infection -> challenge infection

Page 22, line 369: shrimp and -> shrimp, and

Page 23, line 380: L*uminescens -> luminescens*

Page 25, line 414: the type III secretory apparatus -> type III secretory apparatus

Page 25, line 416: better understanding -> a better understanding

Page 26, line 431: Science and -> Science, and

Page 26, line 433: PhD, -> Ph.D.,

Reviewer #2: I acknowledge the authors for addressing my remarks. Few points that still need attention.

In lines 163, 165 and 166 of the revised manuscript there are empty parentheses that either need to be filled or should be deleted.

[Response to the comment]

Page 11, line 162- 165 fixed (Actually, there are not empty parentheses, but the R function(). However, we eliminated the parentheses to avoid the confusion)

In figure 4B the order of the V antigen of the 5 species on the two axes is not the same. Is there a reason for that? If the answer is no please correct the order.
[Response to the comment]

We fixed the error. Now, we aligned them in a correct order.

Reviewer #3: As stated above all my comments have been addressed sufficiently. The new Figure 5 improves the manuscript especially.

[Response to the comment]

We thank you very much for your good advice!
